# A Case of SGLT2 Inhibitor-Induced Euglycemic Diabetic Ketoacidosis

**DOI:** 10.7759/cureus.30106

**Published:** 2022-10-09

**Authors:** Shiavax J Rao, Kaushik Kumar, Nahar Saleh

**Affiliations:** 1 Internal Medicine, MedStar Union Memorial Hospital, Baltimore, USA

**Keywords:** high anion gap metabolic acidosis, sglt-2 inhibitor, euglycemic dka, diabetic keto acidosis, sodium-glucose cotransporter-2 inhibitors

## Abstract

While rare, serious adverse effects including euglycemic diabetic ketoacidosis (EDKA) have been associated with sodium-glucose cotransporter-2 inhibitor (SGLT2i) use. We present an interesting case of SGLT2i-induced EDKA occurring two years after initiation of therapy. Most patients with EDKA recover with prompt recognition and treatment. Patient education about identifying early signs remains a cornerstone of early identification and response to SGLT2i-induced EDKA.

## Introduction

Sodium-glucose cotransporter-2 inhibitors (SGLT2i) are a class of oral diabetic medications that have demonstrated improved cardiovascular and renal outcomes in patients with and without diabetes. While rare, serious adverse effects including euglycemic diabetic ketoacidosis (EDKA) are of concern. We present an interesting case of SGLT2i-induced EDKA occurring two years after initiation of therapy.

## Case presentation

A 57-year-old man presented to the emergency room with generalized weakness, nausea, vomiting, and poor oral intake of one-week duration. His medical history was significant for diabetes mellitus (managed with empagliflozin and metformin). On presentation, he was tachycardic and tachypneic. Physical examination revealed dry mucous membranes and a soft, non-tender abdomen. Initial laboratory diagnostics were remarkable for leukocytosis, elevated blood urea nitrogen, elevated creatinine, low bicarbonate, mild hyperglycemia and high anion gap metabolic acidosis (HAGMA). Lactic acid level, liver function tests, and troponin-I were within normal limits (Table 1). EKG did not reveal acute ischemic changes. He was given 2 liters of normal saline and admitted for management of HAGMA.

**Table 1:** Laboratory diagnostics including serum studies, blood gas and urine studies.

Laboratory Diagnostics	Result	Reference Range
Serum studies		
Leukocyte count	14.4 k/uL	4.0 – 10.8 k/uL
Hemoglobin	14.2 g/dL	12.5 – 16.5 g/dL
Hematocrit	41.3 %	37.5 – 39.5 %
Platelet count	216 k/uL	145 – 400 k/uL
Sodium	133 mmol/L	137 – 145 mmol/L
Potassium	4.9 mmol/L	3.5 – 5.1 mmol/L
Chloride	102 mmol/L	98 – 107 mmol/L
Bicarbonate	12 mmol/L	22 – 30 mmol/L
Blood urea nitrogen	22 mg/dL	9 – 20 mg/dL
Creatinine	1.80 mg/dL	0.66 – 1.50 mg/dL
Glucose	187 mg/dL	65 – 140 mg/dL
Aspartate aminotransferase	10 units/L	0 – 33 units/L
Alanine aminotransferase	10 units/L	10 – 49 units/L
Total bilirubin	0.5 mg/dL	0.3 – 1.2 mg/dL
Direct bilirubin	0.19 mg/dL	0.00 – 0.30 mg/dL
Alkaline phosphatase	88 units/L	46 – 116 units/L
Lipase	46 units/L	12 – 53 units/L
Anion gap	19 mmol/L	5 – 15 mmol/L
Lactic acid	1.6 mmol/L	0.7 – 2.0 mmol/L
Troponin-I (high sensitivity)	46 ng/dL	0 – 53 ng/dL
Ethanol level	< 3 mg/dL	0 – 3 mg/dL
Acetaminophen level	< 2 mcg/mL	10 – 30 mg/dL
Salicylate level	< 3.0 mg/dL	10.0 – 28.9 mg/dL
Blood gas (arterial)		
pH	7.21	7.35 – 7.45
pCO2	23.0 mm Hg	35.0 – 45.0 mm Hg
pO2	163.0 mm Hg	83.0 – 108.0 mm Hg
Bicarbonate	9.2 mmol/L	21 – 28 mmol/L
Urine studies		
Color	Yellow	Yellow
Clarity	Clear	Clear
Specific gravity	1.025	1.005 – 1.030
Glucose	> 1000 mg/dL	Negative
Bilirubin	Negative	Negative
Ketones	> 80 mg/dL	Negative
Blood	Trace	Negative
pH	5.5	5.0 – 8.5
Protein	Trace	Negative/Trace
Urobilinogen	0.2 mg/dL	0.2 – 1.0 mg/dL
Nitrite	Negative	Negative
Leukocyte esterase	Negative	Negative
Amphetamines	None detected	Detectable at 1000 ng/mL
Barbiturates	None detected	Detectable at 200 ng/mL
Benzodiazepines	None detected	Detectable at 200 ng/mL
Cannabinoids	None detected	Detectable at 50 ng/mL
Cocaine	None detected	Detectable at 300 ng/mL
Opiates	None detected	Detectable at 300 ng/mL
Phencyclidine	None detected	Detectable at 25 ng/mL

The patient denied recently consuming ethanol or other toxic alcohols. He endorsed compliance with all home medications. Acetaminophen, ethanol and salicylate levels returned negative. Urinalysis revealed glucosuria and ketonuria (Table 1). Based on clinical history, exam findings and laboratory diagnostics, the patient was diagnosed with EDKA and upgraded to the ICU for further management. He was initiated on a continuous infusion of regular insulin and his intravascular volume loss was corrected with a continuous infusion of dextrose 5% in lactated Ringer’s. Once the anion gap normalized, he was bridged to subcutaneous insulin, and his clinical symptoms drastically improved. He was discharged on basal and short-acting subcutaneous insulin, with planned outpatient follow-up. Empagliflozin was discontinued at the time of discharge. At his follow-up visit, he was doing well without any concerning symptoms. 

## Discussion

SGLT2i are a newer antidiabetic drug class that are recommended as second-line medications for type 2 diabetes mellitus as they enhance excretion and prevent reabsorption of filtered glucose molecules from the proximal renal tubules [1,2]. In recent times, their usage has significantly increased as many large trials have shown significant cardiovascular protective effects [1,3]. In recent guidelines, SGLT2i have also been incorporated as guideline-directed medical therapy for patients with heart failure [4]. EDKA is the presence of ketonemia or ketonuria along with HAGMA with preserved blood glucose levels below 250 mg/dL [5-7]. Not all patients on SGLT2i develop EDKA [5]. In a large multicenter cohort study, the incidence of SGLT2i-induced EDKA was estimated to be 0.25% [5]. Usually a trigger such as infection, pregnancy, fasting, or episodes of vomiting, precipitates a physiologic state of relative insulopenia and increased starvation [7-8].

**Figure 1:**
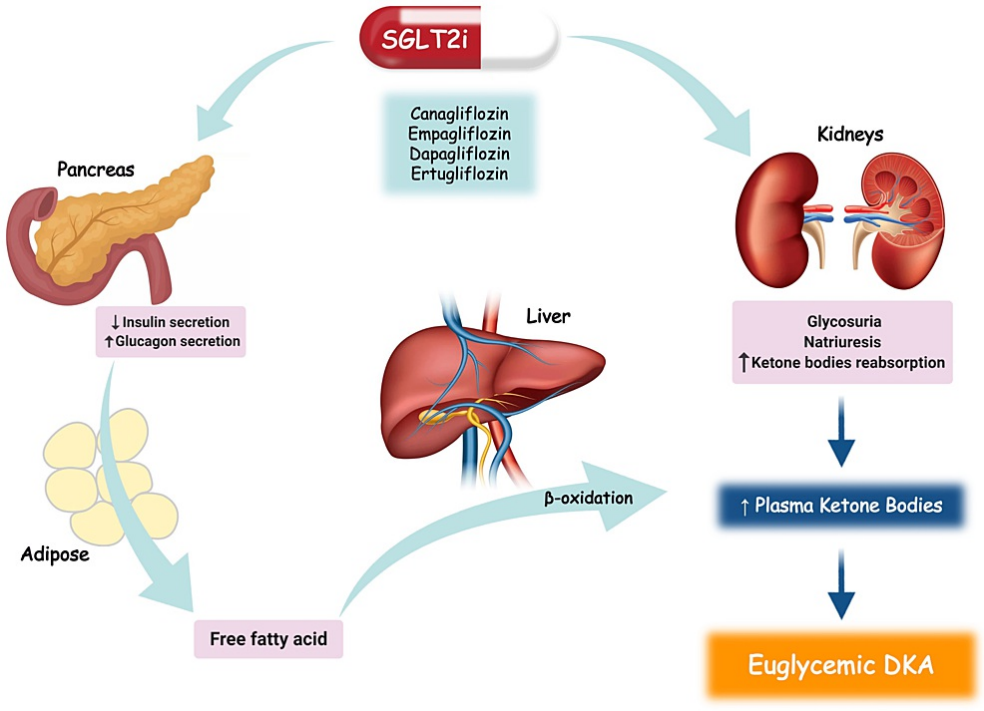
Role of sodium-glucose cotransporter-2 inhibitors in euglycemic diabetic ketoacidosis. SGLT2i: sodium-glucose cotransporter-2 inhibitors, DKA: diabetic ketoacidosis

The elimination of glucose due to SGLT2i mimics a state of carbohydrate starvation and volume depletion. This leads to an increase in the glucagon/insulin ratio. Additionally, SGLT2i are found to directly stimulate the release of glucagon via action on the pancreatic alpha cell, thus promoting hepatic ketogenesis and precipitating ketone formation (Figure 1). The balance between glucosuria and glucagon stimulation results in maintaining a state of euglycemia [6,7]. The Adverse Drug Reaction Probability Scale can be used to assess the probability of the causal relationship between empagliflozin and EDKA. In our patient’s case, the overall score was 7, indicating that there is a probable adverse drug reaction [9].

The management of EDKA is essentially the same as that of DKA, except for timing of administering the dextrose infusion [7-10]. For EDKA, immediately initiating aggressive IV dextrose is of prudence. Any SGLT2i should be held immediately. A favorable prognosis depends on early recognition and screening with serum or urine ketones, even when serum glucose is normal [5-7]. Most patients with EDKA recover with prompt recognition and treatment. Patient education about identifying early signs remains a cornerstone of early identification and response to SGLT2i-induced EDKA [7,10].

## Conclusions

EDKA is a rare yet serious adverse effect of SGLT2i, requiring prompt identification and early initiation of appropriate therapy. Clinicians should have a high index of suspicion for EDKA in patients with euglycemia and HAGMA in the setting of SGLT2i use. Most patients recover with timely recognition and treatment, and patient education about identifying early signs remains a cornerstone of prompt management.
